# The Damaging Effects of Pedunsaponin A on *Pomacea canaliculata* Hemocytes

**DOI:** 10.3390/toxins11070390

**Published:** 2019-07-04

**Authors:** Chunping Yang, Tianxing Lv, Bin Wang, Xiaoyan Qiu, Liya Luo, Min Zhang, Guizhou Yue, Guangwei Qin, Deshan Xie, Huabao Chen

**Affiliations:** 1 College of Agronomy, Sichuan Agricultural University, Chengdu 611130, China; 2 College of Science, Sichuan Agricultural University, Chengdu 611130, China

**Keywords:** *Pomacea canaliculata*, Pedunsaponin A, membrane potential, cytoskeleton, apoptosis, cell cycle

## Abstract

*Pomacea canaliculata* hemocytes are the main functional cells in the immune defense system, and hemocyte destruction disrupts the immune response mechanism of *P. canaliculata*, resulting in abnormal growth, development, reproduction, and even death. Our previous study found that Pedunsaponin A significantly affects *P. canaliculata* hemocyte structure. This study further investigated the damaging effects of Pedunsaponin A on *P. canaliculata* hemocytes. The cell mortality rate results showed that the hemocyte mortality was significantly increased after treatment with Pedunsaponin A, and the mortality rate exhibited a significant positive correlation with treatment time and dose. The membrane potential results showed that the cell membranes of *P. canaliculata* hemocytes exhibited time-dependent membrane depolarization after 40 mg/L Pedunsaponin A treatment. At 36 h, the cell depolarization rate in the Pedunsaponin A treatment group was 41.43%, which was significantly greater than the control group (6.24%). The cytoskeleton results showed that Pedunsaponin A led to disordered and dispersed arrangement of microfilaments and changes in the cytoskeletal structure. The apoptosis and cell cycle results showed that Pedunsaponin A induced apoptosis and influenced the cell cycle to some extent. These results showed that the cell membrane and cytoskeleton of *P. canaliculata* hemocytes were damaged after treatment with Pedunsaponin A, which led to an increase in cell mortality, dysfunction, cell cycle abnormalities and apoptosis. This study provides a foundation for further identification of the site of Pedunsaponin A activity on hemocytes.

## 1. Introduction

*Pomacea canaliculata* is amollusca belonging to invertebrates, and is also known as *Ampullaria gigasl* and apple snail [1]. The immune defense process of invertebrates such as *P. canaliculata* is mainly coordinated by hemocytes involved in cellular immune, hemolymph and immune-related enzymes participating in humoral immunity, since a specific immune system is absent [2,3,4,5]. Hemocytes have intact defense functions, which remove foreign bodies by phagocytosis, packaging, storage and dissolution. Furthermore, external stimulation can activate the phenoloxidase system in hemocytes [6]. Hemocytes are the main functional cells in the *P. canaliculata* immune process, and the immune system is destroyed if hemocytes are disrupted, resulting in abnormal growth and reproduction and even death [7,8].

**Figure 1:**
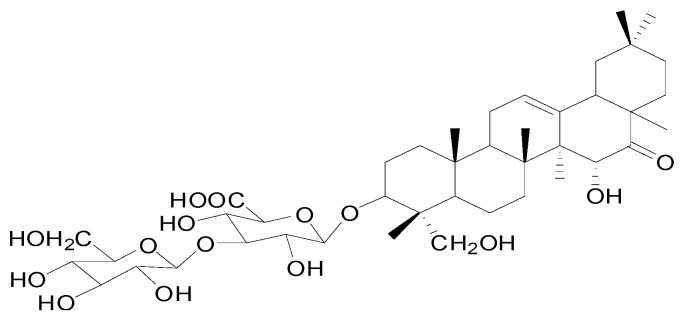
Chemical structure of Pedunsaponin A.

In our previous study, we found that *Pueraria peduncularis* are extremely toxic to *P. canaliculata*; and further identified the active compound as Pedunsaponin A [9] (Figure 1). Through tissue sectioning and electron microscopy observation, we found that Pedunsaponin A has an obvious destructive effect on gill cilia and hemocytes in *P. canaliculata*. The main manifestations of toxicity included the gradual disappearance of gill cilia, aggravated deformation of the cell nucleus and cytoplasm, massive outflow of part of the cytoplasm, dissolution of the nucleus, and obvious effects on the ribosome, mitochondria and other organelles. These results indicate that Pedunsaponin A can significantly affect the structure of hemocytes in *P. canaliculata* which could lead to hemocyte lysis [10]. To determine the damaging effects of Pedunsaponin A on hemocytes, changes in *P. canaliculata* hemocyte mortality, apoptosis, cell cycle, membrane potential and cytoskeleton after treatment with Pedunsaponin A were investigated in this study, which provided a foundation for further identification of the target protein of Pedunsaponin A in hemocytes. 

## 2. Results

### 2.1. The Effects of Pedunsaponin A on the Mortality of P. canaliculata Hemocytes

**Figure 2:**
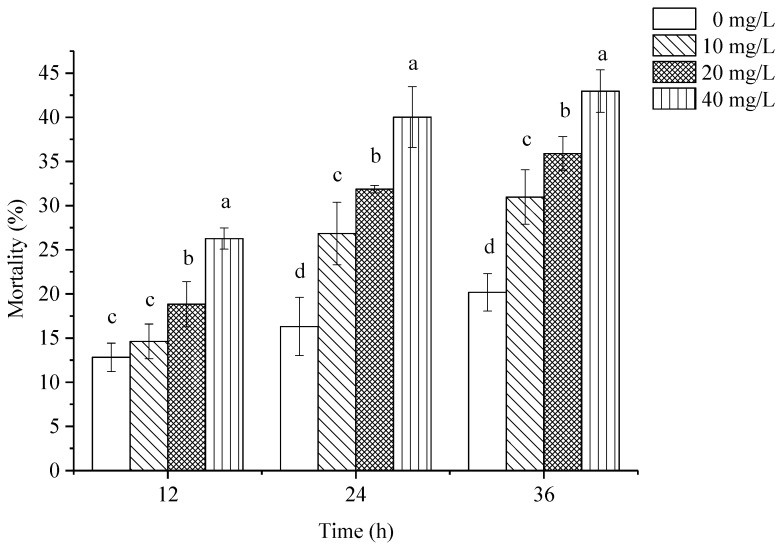
The effects of Pedunsaponin A on the mortality of *P. canaliculata* hemocytes. Note: Values with the different lowercase letters indicate statistically significant differences at the 0.05 probability level (DPS 9.50 (Chinese data processing system), Duncan’s multiple range test).

Hemocyte mortality was analyzed by trypan blue staining. As shown in Figure 2, the mortality of *P. canaliculata* hemocytes increased with increasing treatment concentrations of Pedunsaponin A, and hemocyte mortality was significantly increased with extended treatment time. In addition, hemocyte mortality was time and dose dependent.

### 2.2. The Effects of Pedunsaponin A on the Apoptosis of P. canaliculata Hemocytes

**Figure 3:**
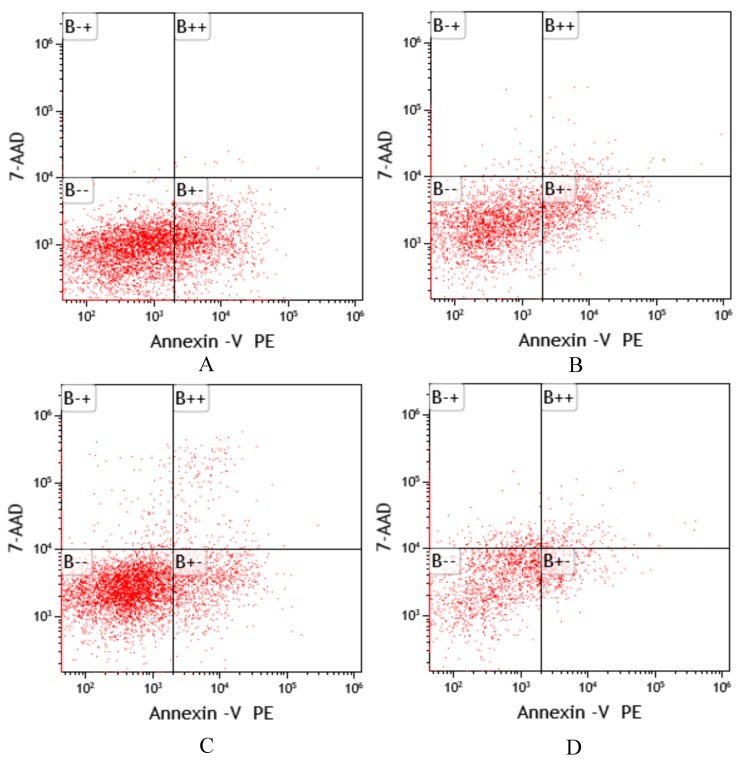
The effects of Pedunsaponin A on the apoptosis of *P. canaliculata* hemocytes at different treatment concentrations detected by flow cytometry. **A**: 0 mg/L (control); **B**: 10 mg/L; **C**: 20 mg/L; **D**: 40 mg/L. B−−: normal cells; B−+: dead cells; B++: earlier stage of apoptotic cells; B+−: later stage of apoptotic cells.

**Figure 4:**
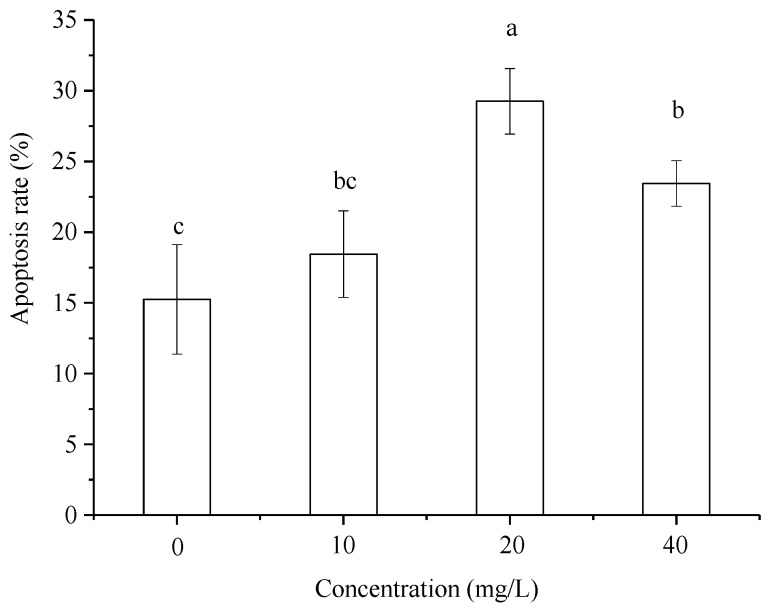
The apoptosis rate of *P. canaliculata* hemocytes after treatment with Pedunsaponin A at different concentrations for 36 h. Note: Values with the different lowercase letters indicate statistically significant differences at the 0.05 probability level (DPS 9.50, Duncan’s multiple range test).

The results of *P. canaliculata* hemocyte apoptosis are shown in Figure 3, and the analyzed data are shown in Figure 4. The results indicated that after treatment with different concentrations of Pedunsaponin A, the proportion of hemocyte apoptosis increased first and then decreased. Combined with the results of hemocyte mortality, this phenomenon may be due to low mortality since the lower concentration of Pedunsaponin A mainly caused cell apoptosis. However, the mortality of hemocytes increased significantly and the number of apoptotic cells decreased at high concentrations, which directly led to necrosis. 

### 2.3. The Effects of Pedunsaponin A on the Cell Cycle of P. canaliculata Hemocytes

**Figure 5:**
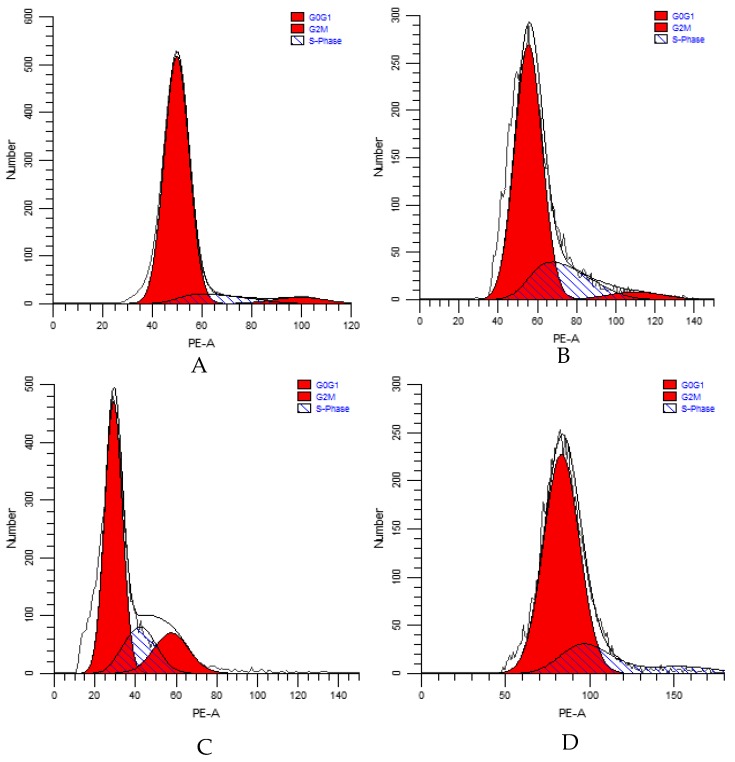
The effects of Pedunsaponin A on the cell cycle of *P. canaliculata* hemocytes at different treatment concentrations detected by flow cytometry. **A**: 0 mg/L (control); **B**: 10 mg/L; **C**: 20 mg/L; **D**: 40 mg/L. Note: G0G1 indicates the DNA pre-synthesis phase. G2M indicates prophase and the mitotic phase, and S indicates the DNA synthesis phase.

**Figure 6:**
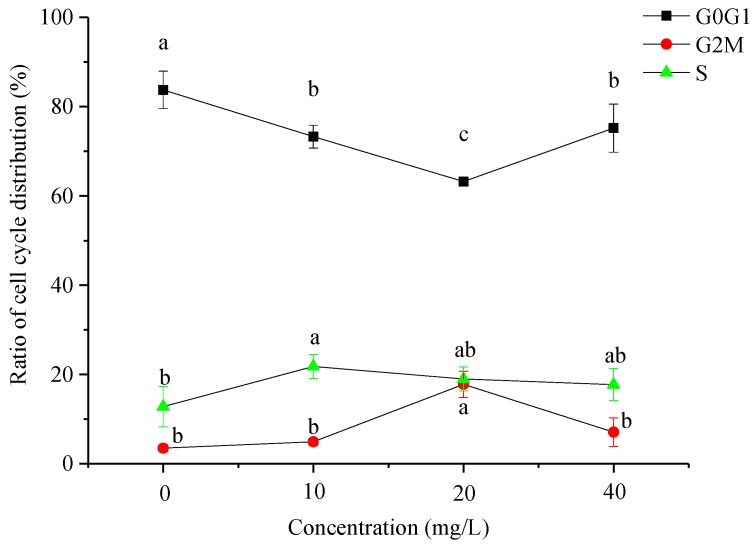
The ratio of cell cycle distribution of *P. canaliculata* hemocytes after treatment with Pedunsaponin A at different concentrations for 36 h. Note: Values with the different lowercase letters indicate statistically significant differences at the 0.05 probability level (DPS 9.50, Duncan’s multiple range test).

*P. canaliculata* were treated with 10 mg/L, 20 mg/L, and 40 mg/L Pedunsaponin A for 36 h and the cell cycle distribution was analyzed by flow cytometry, as shown in Figure 5. The analyzed data are shown in Figure 6. The proportion of hemocytes in the G0/G1 phase in the control group was significantly greater than that in the treatment group. The proportion of hemocytes in the G0/G1 phase in the treatment group decreased following treatment with different concentrations of Pedunsaponin A, while the proportion of hemocytes in the S and G2/M phases increased, indicating that cell proliferation was accelerated. The proportion of hemocytes in the G0/G1 phase was the lowest under 20 mg/L Pedunsaponin A treatment and was significantly less than that in the control group (*P* < 0.05), while the hemocytes in the G2/M and S phases were increased under 20 mg/L Pedunsaponin A treatment compared with the control group. The abovementioned results indicated that hemocytes under the lower treatment concentration of Pedunsaponin A exhibited a change in the cell cycle and accelerated cell proliferation. Under the relatively high treatment concentration of Pedunsaponin A, the hemocytes exhibited necrosis and a low rate of cell proliferation, and the proportion of hemocytes in the G2/M and S phases increased, but there was no significant difference compared with the control group.

### 2.4. The Effects of Pedunsaponin A on the Membrane Potential of P. canaliculata Hemocytes

**Figure 7:**
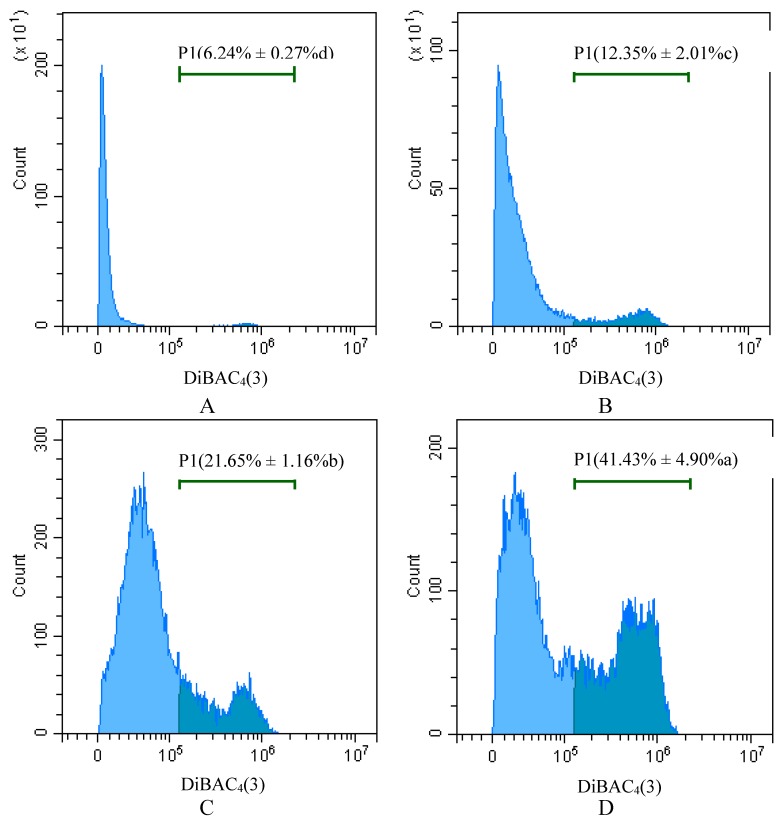
The effects of treatments with 40 mg/L Pedunsaponin A for different treatment times on the membrane potential of *P. canaliculata* hemocytes. **A**: water (control); **B**: 12 h; **C**: 24 h; **D**: 36 h. Note: P1 indicates the ratio of membrane depolarized cells.

DiBAC_4_(3) staining and flow cytometry were used to analyze the change in hemocyte membrane potential after treatment with Pedunsaponin A. According to the results shown in Figure 7, compared with the control group (6.24%), the depolarization of *P. canaliculata* hemocytes was increased to 12.35%, 21.65%, and 41.43% after treating *P. canaliculata* with 40 mg/L Pedunsaponin A for 12 h, 24 h, and 36 h, respectively. This result indicated that Pedunsaponin A significantly affected the hemocyte membrane potential, and the membrane showed time-dependent depolarization.

### 2.5. The Effects of Pedunsaponin A on the Cytoskeleton of P. canaliculata Hemocytes

**Figure 8:**
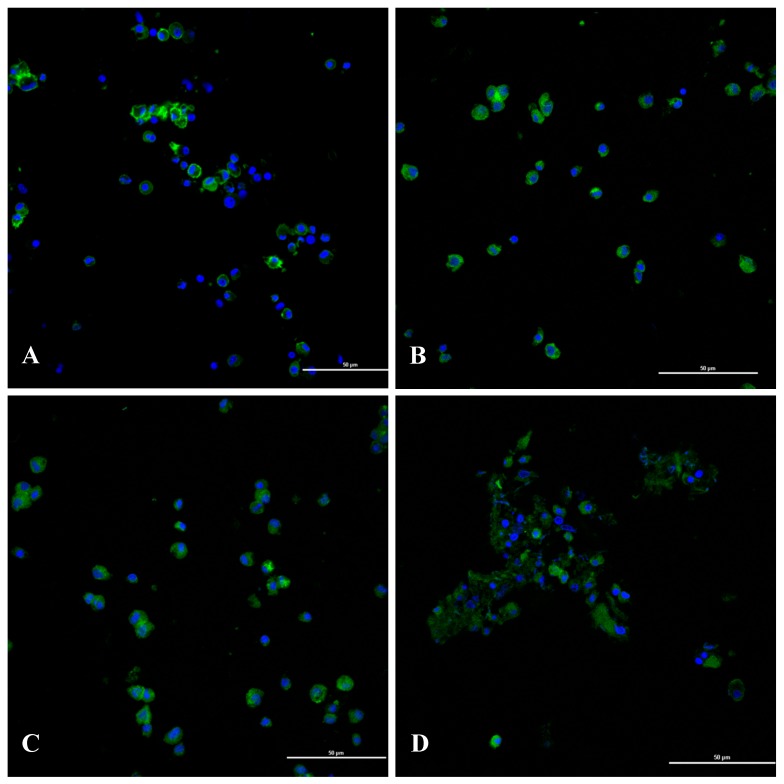
The effects of treatment with 40 mg/L Pedunsaponin A for different treatment times on the cytoskeleton of *P. canaliculata* hemocytes. **A**: water (Control); **B**: 12 h; **C**: 24 h; **D**: 36 h. Scale bars: 50 µm.

Changes in the hemocyte cytoskeleton were observed under laser scanning confocal microscopy after treating *P. canaliculata* with Pedunsaponin A. The results showed that the cytoskeleton microfilaments in the hemocytes of the control group were mainly distributed on the cell membrane and in the cytoplasm, and the membrane was surrounded with obvious zonal fluorescence. As the Pedunsaponin A treatment time increased, fluorescence on the membrane decreased, leading to a disordered and dispersed arrangement of the microfilaments, and the structure of the microfilaments was eventually transformed. These results showed that Pedunsaponin A significantly affected the distribution of microfilaments and destroyed the cytoskeletal structure in hemocytes. In addition, the influence of Pedunsaponin A on the cytoskeleton was time-dependent (Figure 8).

## 3. Discussion

The cell membrane is an important site for exchange of information, substances and energy between cells and the external environment, and the cell membrane plays an important role in resisting external stimuli and preventing cell damage [11,12]. The integrity of the cell membrane is the basis for the normal function of cells, and the toxic effect of drugs on cells is closely related to changes in membrane structure [13]. Trypan blue staining was used in this experiment to analyze the mortality of hemocytes after treatment with Pedunsaponin A. The results showed that the activity of hemocytes was obviously decreased after Pedunsaponin A treatment, and hemocyte mortality exhibited a significant positive correlation with treatment time and dose. These results indicated that if the cell membrane of hemocytes was damaged and the integrity of the cell membrane was lost, the membrane permeability eventually increased. To verify the effects of Pedunsaponin A on hemocytes, a DiBAC_4_(3) membrane potential fluorescence probe was combined with flow cytometry to analyze the change in membrane potential after treatment with 40 mg/L Pedunsaponin A for 12 h, 24 h, and 36 h. We observed that the cell membranes of *P. canaliculata* hemocytes exhibited time-dependent membrane depolarization, and the higher the concentration of Pedunsaponin A, the more serious the depolarization. Cell membrane depolarization causes the cations outside of the cell membrane to flow into the cell, which changes the internal and external pressure of the cell and breaks the balanced distribution of ions on both sides of the cell membrane, leading to changes in the physiological and biochemical state of the cell and destruction of hemocytes. This is an important reason for the increase in hemocyte mortality. 

The cytoskeleton is a skeletal network structure consisting of proteins that plays an indispensable part in maintaining the basic morphology of cells and participates in intracellular and extracellular signal transmission, cell division and energy conversion. This mechanism ensures oriented trans-shipment in the intracellular regions of various alveoli and organelles [14,15,16,17,18], and normal physiological functions are disrupted naturally when the cytoskeleton is affected [19]. Zhu et al. [20] found that *Pteromalus puparum* parasitized on cabbage butterflies inhibited the transcription level of the host cytoskeletal protein genes and inhibited the hemocytic immune response. In the present study, we found that the fluorescence intensity of the microfilaments on the cell membrane was gradually weakened, and the arrangement and dispersion of the microfilaments were disordered after treating *P. canaliculata* with Pedunsaponin A. Eventually, the cytoskeleton and cell structure were destroyed. 

The normal function of a cell is affected if the cell membrane and cytoskeleton are damaged, and cell dysfunction leads to abnormal cell cycle and apoptosis. According to the cell apoptosis results after treating *P. canaliculata* hemocytes with Pedunsaponin A, the ratio of apoptosis first increased and then decreased as the concentration of Pedunsaponin A increased. Combined with the cell mortality results, we found that hemocyte mortality was very low because treated *P. canaliculata* were treated with relatively low concentrations of Pedunsaponin A, and cell apoptosis was the main factor in cell death. Under high concentrations of Pedunsaponin A, the mortality of hemocytes was significantly increased, the number of apoptotic cells decreased, and the hemocytes trended toward necrocytosis. The cell apoptosis results showed that the ratio of hemocytes in the G0/G1 phase was decreased, while the ratio of hemocytes in the S and G2/M phases was increased, and cell proliferation was accelerated after treatment with Pedunsaponin A, indicating that replication of *P. canaliculata* hemocyte genetic material was affected by Pedunsaponin A treatment. These results demonstrated that Pedunsaponin A can induce apoptosis and influence the cell cycle to some extent.

According to the results of our study, we inferred that *P. canaliculata* hemocytes are the potential targets of Pedunsaponin A, as the cell membrane and cytoskeleton of hemocytes were severely damaged after treatment with Pedunsaponin A, causing the increase in membrane permeability which further leads to the change in membrane potential. All of these changes affected the normal function of hemocytes and caused the increase of hemocyte mortality, eventually resulting in the death of *P. canaliculata*. This study provides a foundation for further identification of the site of Pedunsaponin A activity.

## 4. Materials and Methods

### 4.1. Materials

*P. canaliculata* were collected from Chengdu City, Sichuan Province, China (30.71° N, 103.87° E), in July 2015. We maintained the snails for at least 5 days to acclimatize them to the laboratory conditions. The snails were housed in 50 L plastic aquariums at room temperature with natural light conditions and without artificial aeration. Water and dead snails were removed daily from these aquariums. The snails were fed fresh cabbage leaves. The weight of the snails used in this study was 5 ± 0.5g. 

Roots of *P. peduncularis* were collected from wild populations in Yaan City, Sichuan Province, China (29.90° N, 102.92° E). Pedunsaponin A was isolated from the root extracts of *P. peduncularis* by the Biorational Pesticide Laboratory of Sichuan Agricultural University. The purity of Pedunsaponin A was greater than 98%.

### 4.2. Methods

#### 4.2.1. Preparation of Hemocyte Suspension

The shell around the heart of both treated and control *P. canaliculata* were removed by dissecting scissors, and a 1 mL disposable syringe was used to suction hemolymph into a 1.5 mL centrifuge tube preloaded with anticoagulant solution (1% heparin sodium, Bejing Solarbio Science & Technology Co. Ltd., Beijing, China). The sediment was retained and the supernatant was discarded after 5 min of centrifugation (940.5× *g*, Beckman Coulter, Brea, CA, USA). PBS solution (pH 7.2–7.4, Bejing Solarbio Science & Technology Co. Ltd, Beijing, China) was added to wash the deposits. Subsequently, the above steps were repeated once more, and the hemocyte deposits were then re-suspended.

#### 4.2.2. Mortality Analysis of Hemocytes

Ten adult *P. canaliculata* were allocated to each group and treated with 10 mg/L, 20 mg/L, and 40 mg/L Pedunsaponin A for 12 h, 24 h, and 36 h respectively. The method of hemolymph extraction from the atrial appendage and hemocyte suspension was the same as the method mentioned in Section 4.2.1. The control group was treated with water. For each experiment, three independent replicates were conducted.

According to a method reported by Alex Karnowski [21], the cell density was adjusted to 1 × 10^6^ cells/mL and the cell suspension mixed with 0.4 % trypan blue solution (Bejing Solarbio Science & Technology Co. Ltd, Beijing, China) at volume ratio of 9:1. Within 3 min, a blood cell counting board was used to count live and dead cells under a light microscope (10 × 40 magnification). The dead cells were stained blue, and the live cells were colorless and transparent. Hemocyte mortality was calculated by the following formula: mortality rate (%) = total number of dead cells/(total number of living cells + total number of dead cells) × 100%.

#### 4.2.3. Apoptosis Analysis of Hemocytes

Ten adult *P. canaliculata* were allocated to each group and treated with 10 mg/L, 20 mg/L, and 40 mg/L Pedunsaponin A for 36 h. The method of hemolymph extraction from the atrial appendage and hemocyte suspension was the same as the method mentioned in Section 4.2.1. The control group was treated with water. For each experiment, three independent replicates were conducted. 

A total of 100 µL of hemocyte suspension and 5 µL annexin V-PE detection reagent (BioVision, San Francisco, CA, USA) were added to the flow tube and mixed evenly at 4 °C for 15 min protected from light, and 2 mL binding buffer was added to the flow tube to re-suspend the cells. After centrifugation for 15 min (67× *g*) and discarding the supernatant, 450 µL of binding buffer was added to the re-suspended cells [22]. Apoptosis was immediately detected by flow cytometry, and the results were analyzed by the Kaluza software (1.5a for Windows, Beckman Coulter).

#### 4.2.4. Cell Cycle Analysis of Hemocytes

Ten adult *P. canaliculata* were allocated to each group and treated with 10 mg/L, 20 mg/L, and 40 mg/L Pedunsaponin A for 36 h. The method of hemolymph extraction from the atrial appendage and hemocyte suspension was the same as the method mentioned in Section 4.2.1. The control group was treated with water. For each experiment, three independent replicates were conducted. 

According to a previous method reported by Zhao et al. [23], 10 µL propidium iodide (PI) solution and 10 µL RNase A solution (Leagene, Beijing, China) were added to 0.5 mL dyeing buffer and mixed well, and 0.5 mL of prepared PI staining solution was added to the cell samples and gently mixed to re-suspend the cells. After 30 min of incubation in a water bath at 37 °C in the dark, the samples were preserved in ice in the absence of light. Cell cycle analysis was conducted by flow cytometry at an excitation wavelength of 488 nm, and the data were analyzed by the Kaluza software.

#### 4.2.5. Membrane Potential Examination of Hemocytes

Ten adult *P. canaliculata* were allocated to each group and treated with 40 mg/L Pedunsaponin A for 12 h, 24 h, and 36 h. The method of hemolymph extraction from the atrial appendage and hemocyte suspension was the same as the method mentioned in Section 4.2.1. The control group was treated with water. For each experiment, three independent replicates were conducted.

Then, 5 µmol/mL DiBAC_4_(3) (Bejing Solarbio Science & Technology Co. Ltd, Beijing, China) was added to 1 mL hemocyte suspension, and flow cytometry was used to detect membrane potential after reacting for 30 min in the dark. The flow cytometry detection parameters included an excitation wavelength of 488 nm and an emission wavelength of 520 nm [24].

#### 4.2.6. Cytoskeletal Analysis of Hemocytes

Ten adult *P. canaliculata* were allocated to each group and treated with 40 mg/L Pedunsaponin A for 12 h, 24 h, and 36 h. The method of hemolymph extraction from the atrial appendage was the same as the method mentioned in Section 4.2.1. The control group was treated with water. For each experiment, three independent replicates were conducted.

A total of 40 μL of hemolymph was dropped onto a slide to make a blood smear and then dried at room temperature. Subsequently, the hemocyte blood smears were fixed with 4% paraformaldehyde solution for 30 min and washed three times with PBS buffer solution for 5 min for each wash. At room temperature, 0.5% Triton X-100 solution was used to treat the hemocyte blood smears for 5 min after acetone (−20 °C) was dehydrated swiftly. Then, PBS buffer solution was used to wash the blood smear three times for 5 min for each wash. Fluorescein isothiocyanate (FITC)-labeled phalloidin (100 µL, Bejing Solarbio Science & Technology Co. Ltd, Beijing, China) was added to the samples, and the samples were incubated in the dark for 2 h. Then PBS buffer solution was used to wash the blood smear three times for 5 min for each wash. Next, 200 μL 4′,6-diamidino-2-phenylindole (DAPI) dye solution (Bejing Solarbio Science & Technology Co. Ltd, Beijing, China) was added to the blood smears, incubated at room temperature for 10 min in the dark, and washed 3 times with PBS buffer solution for 5 min for each wash. A drop of Fluoromount-G^TM^ water soluble seal (Qcbio S&T, Shanghai, China) was dropped onto the slide, and a cover slip was placed on the slide. Fluorescence was observed under a laser scanning confocal microscope (Nikon, C2, Beijing, China). The FITC excitation wavelength was 496 nm, and the emission wavelength was 516 nm. The DAPI excitation wavelength was 364 nm and emission wavelength was 454 nm. FITC-labeled phalloidin solution dyed the microfilaments green, and DAPI dyed the nuclei blue.
